# Progressive Exercise Training After Aortic Dissection in a Recreational Athlete

**DOI:** 10.1016/j.jaccas.2025.103998

**Published:** 2025-07-16

**Authors:** Ashley E. Carlisle, Leah G. Gutzwiller, Brian P. Shapiro, Elizabeth H. Dineen, Kevin G. Tayon, Kiyan Heybati, Craig P. Stone, Brandyn M. Rader, Bryan J. Taylor

**Affiliations:** Department of Cardiovascular Diseases, Mayo Clinic, Jacksonville, Florida, USA

**Keywords:** aortic dissection, exercise prescription, exercise training

## Abstract

**Objective:**

Structured exercise training (ExT) has been shown to improve cardiorespiratory fitness and health-related quality of life and is safe in patients after surgery for type A aortic dissection (AD).

**Key Steps:**

We detail the implementation of progressive ExT in a 56-year-old man former recreational triathlete 16 months after emergency repair of a type I AD, who had a residual descending aortic dissection. A home-based individualized ExT plan incorporated low- to moderate-intensity aerobic and resistance exercise with progression to interval training.

**Potential Pitfalls:**

Exercise is often limited or withheld in patients with previous AD because of concern that sudden blood pressure elevation may increase the risk of recurrent dissection or propagation of existing dissection. Patients, once medically optimized, need proper education to safely self-monitor and adhere to ExT.

**Take-Home Message:**

This case illustrates the potential feasibility, benefit, and safety of exercise after AD, and demonstrates the need to establish evidence-based guidelines.

## Visual Summary

**Figure:**
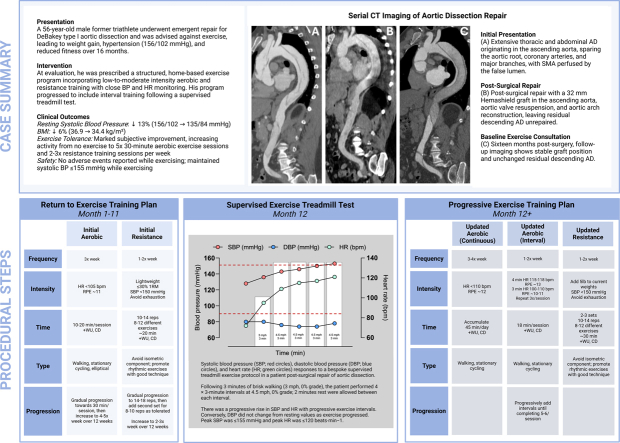

Acute aortic dissection (AD), characterized by an intimal tear in the aorta allowing blood to penetrate the aortic wall layers, represents a medical emergency with mortality rates of up to 0.5% per hour within the first 48 hours if left untreated. Exercise recommendations for patients after AD represents a clinical dilemma. Although regular physical activity and exercise training (ExT) can lower heart rate (HR), blood pressure (BP), and body weight, it has been presumed that sudden exercise-related elevations in BP may transiently increase the risk of recurrent aortic pathology. While it has been suggested that intense prolonged isometric exercise should be avoided after AD because of the risk of recurrence or progression, recent guidelines recommend exercise at a maximal intensity of 3-5 METs.^1^^,^^2^ However, the exact threshold of safe exercise intensity remains unclear, often leaving clinicians uncertain regarding exercise clearance.^3^ Although 58% of providers advised patients to self-monitor BP and HR during physical activity, there was significant inconsistency regarding safe upper limits.^3^ Furthermore, 71% of patients expressed a desire for clear and specific recommendations on safe physical activities after AD.^4^ Yet current guidelines lack specific parameters such as BP or HR thresholds during exercise because of limited data; guidelines suggest personalized recommendations based on factors such as underlying aortic pathology, diameter, growth rate, and residual unrepaired dissection, and generally recommend against participation in competitive sport.^1^^,^^2^ Athletes accustomed to high-intensity exercise may experience a profound loss in physical capacity affecting physical and mental recovery. Given the documented benefits of regular exercise,^1^ there is clear clinical need for personalized exercise plans developed through shared decision making (SDM) between patients and providers. This case report outlines the successful application of a progressive ExT program through SDM in a 56-year-old former recreational triathlete after surgical repair of an ascending AD.

**Take-Home Messages**

• In selected patients, structured individualized exercise training may be safely implemented after aortic dissection with potential benefits in cardiovascular health and functional recovery.
• More investigation is needed to assess safety and efficacy of exercise training across a larger patient population, provide insights into longer-term outcomes, and ultimately establish further evidence-based guidelines for exercise in this population.

## Case Summary

**Figure 1:**
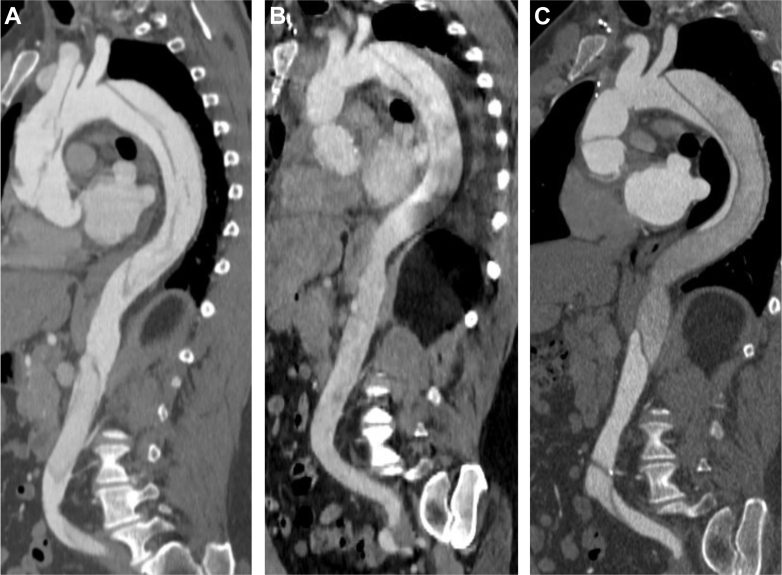
Computed Tomography Imaging of a 56-Year-Old Man With Aortic Dissection (A) Initial presentation shows an extensive thoracic and abdominal AD originating in the ascending aorta, sparing the aortic root, coronary arteries, and major branches, with super mesenteric artery perfused by the false lumen. (B) Postsurgical repair with a 32-mm Hemashield graft in the ascending aorta, aortic valve resuspension, and aortic arch reconstruction, leaving residual descending AD unrepaired. (C) Sixteen months after surgery, follow-up imaging shows stable graft position and unchanged residual descending AD.

A 56-year-old male former recreational triathlete with no significant past medical history was admitted to his local emergency department with epigastric and back pain. On initial presentation, BP was 176/73 mm Hg and HR was 44 beats/min. Nongated computed tomography (CT) chest angiography revealed an extensive combined thoracic and abdominal AD originating in the ascending aorta at the sinotubular junction and extended to the aortic arch, descending thoracic aorta, abdominal aorta, terminating within the left iliac artery. The super mesenteric artery was perfused by the false lumen of the AD. The aortic root, coronary arteries, branches of the aortic arch, and left iliac artery were spared. The ascending aorta was 4.9 cm in maximal dimension without evidence of rupture (Figure 1A). Emergency surgical repair was performed using a 32-mm Hemashield graft in the ascending aorta, aortic valve resuspension to preserve the native valve, and aortic arch reconstruction with Hemashield patch. No intervention was done on the remaining AD extending from the aortic arch distal to the left subclavian ostium to the left iliac artery (Figure 1B). Beta-blocker, angiotensin-converting enzyme inhibitor, calcium channel blocker, and statin therapy were initiated.

Sixteen months after surgical repair, the patient presented to Mayo Clinic Florida for evaluation and exercise guidance. Despite improved diet and attempts to optimize pharmacotherapy, he reported weight gain (+26 lb, body mass index from 33.2 to 36.9 kg/m^2^), resting hypertension (156/102 mm Hg), fatigue, and exertional intolerance after being counseled to limit exercise after AD. His significant apprehension regarding physical activity, driven by concerns about the residual dissection, further compounded his physical deconditioning. Physical examination was otherwise unremarkable.

Repeated CT chest angiography showed stable position of the ascending aortic graft and residual but stable unrepaired descending aortic dissection as previously described, with no change compared with immediate postoperative imaging (Figure 1C). The patient was referred for exercise consultation to guide safe return to exercise.

## Procedural Steps

### Initial consultation and exercise plan

After extensive review of the patient’s history, and based on the best available guidelines, the patient was prescribed a low-intensity progressive aerobic and resistance ExT plan to gradually reintroduce physical activity. The focus was on aerobic and muscular reconditioning, with the goal of improving cardiovascular fitness and addressing cardiovascular risk factors. SDM guided the establishment of safe HR, BP, and rating of perceived exertion (RPE) limits along with goals for progression.

**Figure 2:**
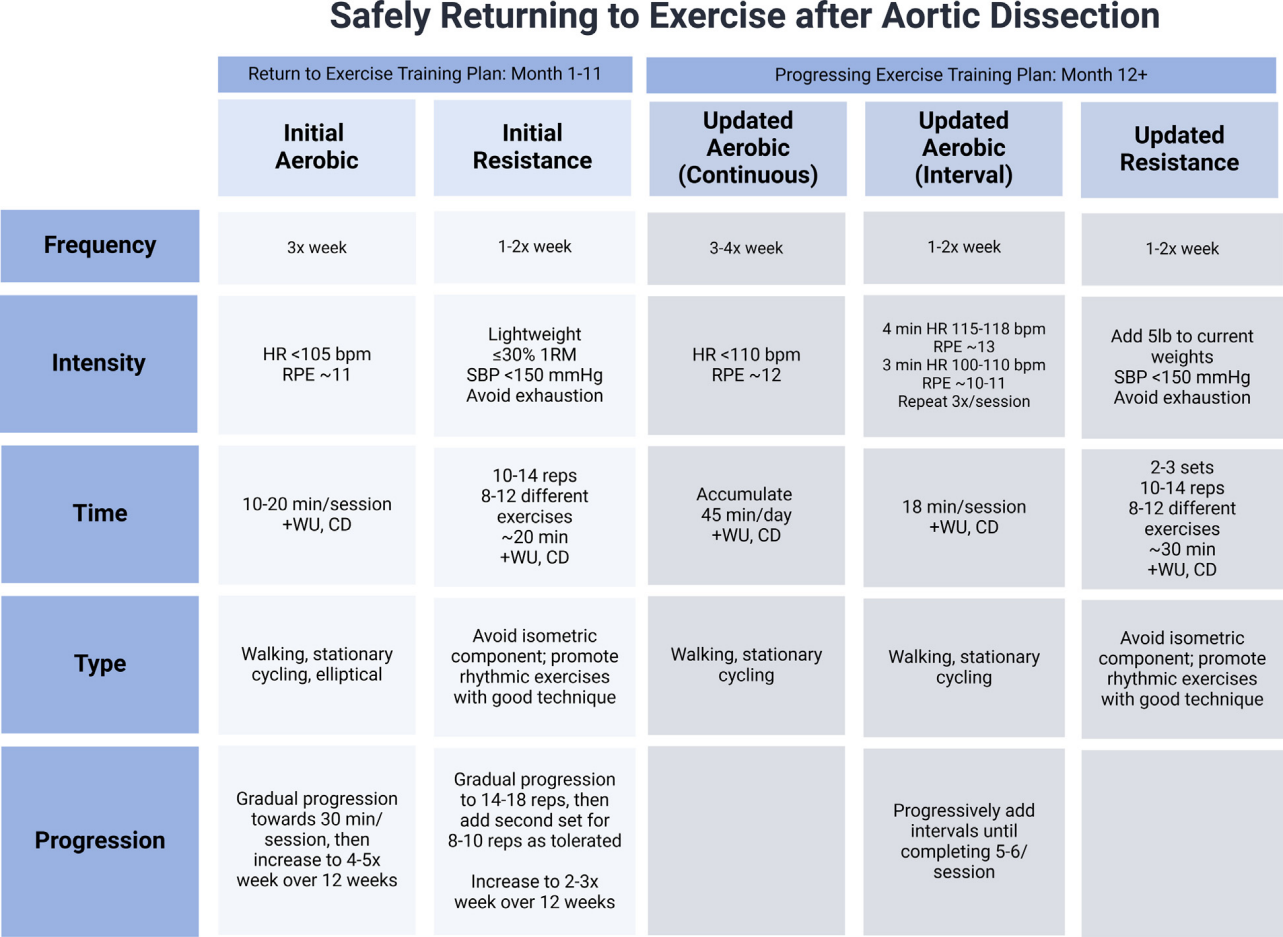
Progressive Exercise Training Plan for Post-Aortic Dissection Rehabilitation The plan outlines initial (left) and updated (right) aerobic and resistance training regimens, including frequency, intensity, time (including warm-up [WU] and cool-down [CD]), type, and progression, with specific parameters for heart rate (HR), blood pressure (BP), and rating of perceived exertion (RPE) to promote safety.

The initial plan (Figure 2) gradually increased aerobic exercise duration from 15 to 30 minutes per session and frequency from 4 to 5 days per week over the first 12 weeks. Resistance training was prescribed twice weekly, targeting major muscle groups with 1 set of 10-14 repetitions of 8-12 different exercises using light resistance (<30% 1-repetition maximum). The patient was guided to prioritize dynamic exercises with good form (eg, bicep curls, triceps kickbacks), avoiding substantial isometric exercises and breath-holding (eg, squats, planks) and task-failure inducing fatigue. The repetitions per exercise progressed to 14-18 before a second set of 8-10 repetitions was added as tolerated over the first 12 weeks. For all exercise, the patient was instructed to monitor BP and ensure that systolic BP remained ≤150 mm Hg, a target established based on laboratory monitoring during exercise sessions to achieve lower and safer BP levels.

### Follow-up testing and plan progression

**Figure 3:**
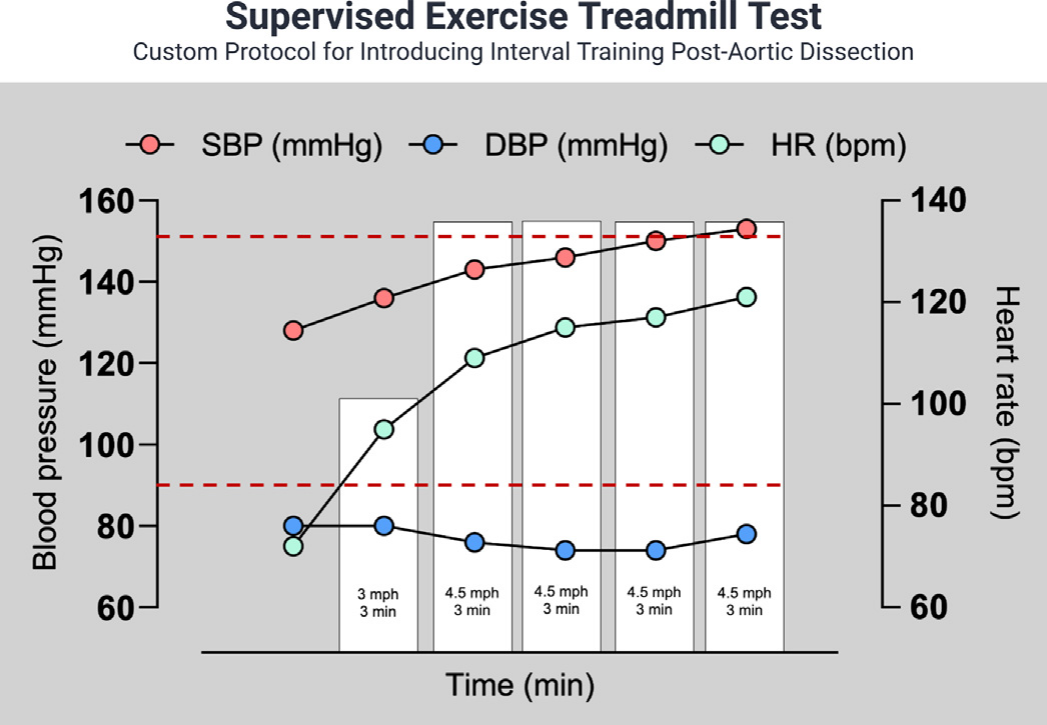
Supervised Exercise Treadmill Test Systolic blood pressure (SBP) (red circles), diastolic blood pressure (DBP) (blue circles), and heart rate (HR) (green circles) responses to a bespoke supervised treadmill exercise protocol in a patient after surgical repair of aortic dissection. White bars signify each interval, with the maximum speed and total interval time noted for each. Red dotted lines show the set parameters for SBP (≤150 mm Hg) and HR (<120 beats/min). After 3 minutes of brisk walking (3 mph, 0% grade), the patient performed 4 × 3-minute intervals at 4.5 mph, 0% grade. Two minutes of rest was allowed between each interval. There was a progressive rise in SBP and HR with progressive exercise intervals. Conversely, DBP did not change from resting values as exercise progressed. Peak SBP was ≤155 mm Hg and peak HR ≤120 beats/min.

The patient self-reported “good” to “excellent” adherence to the prescribed plan without complication or adverse event at follow-up 11 months later. Notable improvements were observed in BMI (36.9 to 34.4 kg/m^2^) and resting BP (156/102 to 135/84 mm Hg). However, he felt his training had “plateaued” and sought advancing his exercise regimen. Through SDM, the patient underwent a supervised treadmill exercise test to assess the physiologic response to short intermittent periods of light jogging (Figure 3). The patient tolerated the test without symptoms and was discharged from the laboratory in stable condition.

**Visual Summary:**
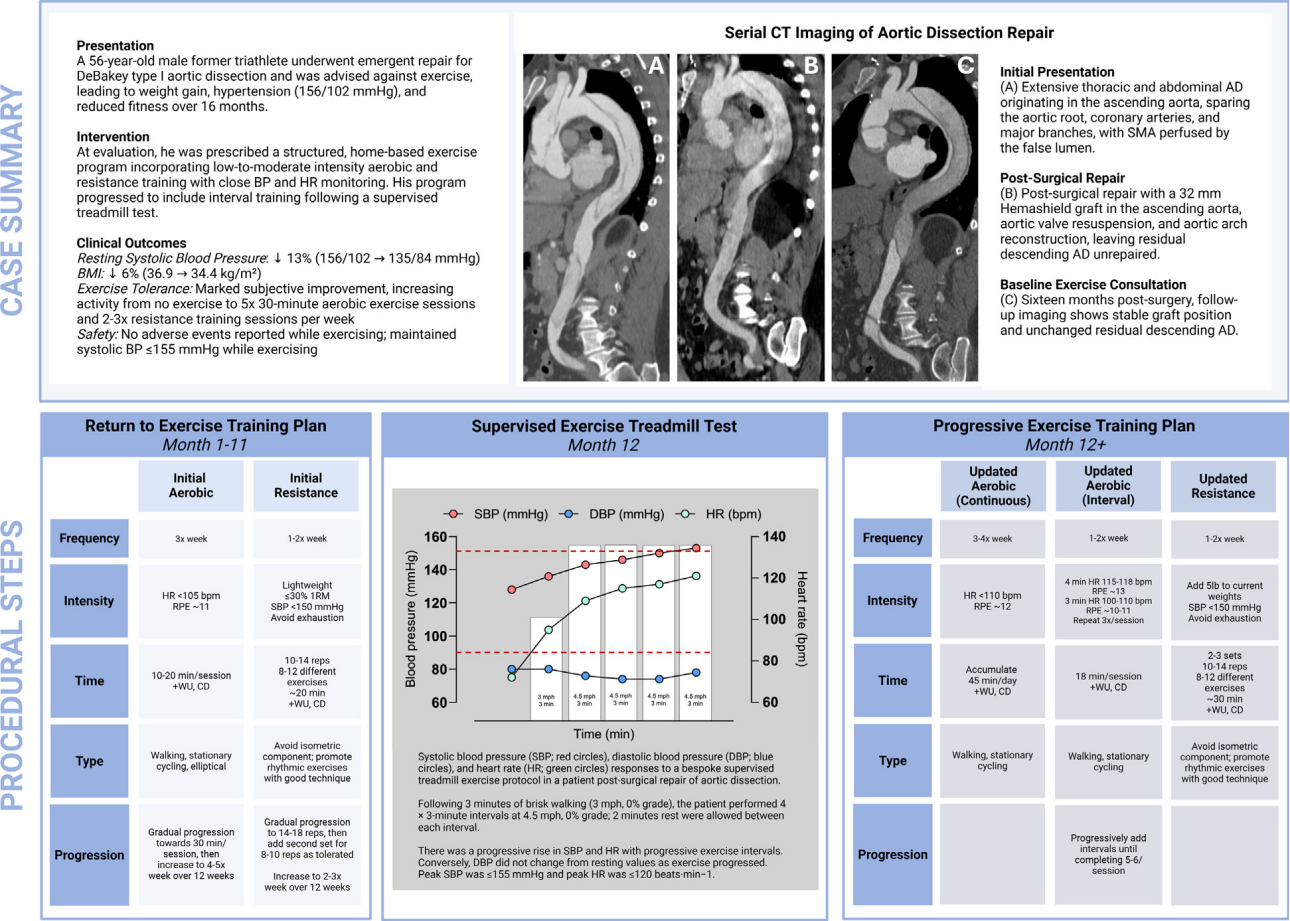
Case Summary, Serial Imaging, and Procedural Steps of Return to Exercise 1 RM = 1-repetition maximum; BMI = body mass index; BP = blood pressure; CD = cool-down; CT = computed tomography; DBP = diastolic blood pressure; HR = heart rate; RPE = rating of perceived exertion; SBP = systolic blood pressure; WU = warm-up.

Given the apparent feasibility and lack of adverse events during moderate-to-hard intervals of aerobic exercise, the training plan was adjusted to include interval aerobic training with 4-minute intervals at 115-118 beats/min (RPE ∼13) and 3-minute recoveries at 100-110 beats/min (RPE ∼10-11), repeated 3 to 5 times per session. Resistance exercises progressed to 35% 1-repetition maximum for 2-3 sets of 10-14 repetitions (Figure 2).

This structured progressive approach to ExT facilitated measurable improvements in cardiovascular risk factors, including a 13% reduction in resting systolic BP and 6% decrease in weight, with self-reported improvements in exercise tolerance. These results suggest that individualized exercise plans can contribute to improvements in cardiovascular health after AD without clinically adverse events. Follow-up CTA imaging was not available after the exercise program to confirm the stability of the aorta. While clinical stability was observed throughout the training period, the lack of imaging limits the ability to definitively assess structural outcomes.

## Potential Pitfalls

Despite recent expert recommendations,^1^^,^^2^ ExT after AD remains a contentious clinical topic. Indeed, engagement in physical activity tends to decline after AD.^4^ While not underpinned by compelling clinical evidence or robust trials, concerns about sudden BP surges increasing the risk of recurrent dissection or aortic rupture underscores the hesitancy in prescribing exercise and the desire for ongoing monitoring. Emerging evidence suggests dynamic aerobic and resistance exercises, such as brisk walking or cycling, at intensities described as “fairly light” to “somewhat hard” are generally safe, provided that patients avoid isometric exertion, breath-holding, and task-failure fatigue.^5^^,^^6^ Cardiopulmonary exercise testing is an emerging, potentially safe tool to personalize exercise prescriptions in this population, enabling moderate exercise intensities to be tailored to each patient’s aerobic capacity and functional status.^7^

The initial exercise evaluation is particularly important for patient selection and education. Candidates should demonstrate stable aortic anatomy confirmed by imaging without heritable disorders and be capable of self-monitoring symptoms and adjusting exercise intensity to avoid overexertion. Patient education is vital in home-based programs, because patients must be capable of accurately self-monitoring BP and HR during exercise. This self-monitoring enables them to adjust exercise intensity in real time to minimize the risk of overexertion. Importantly, adherence to the exercise plan and avoidance of injury are shared responsibilities, requiring active patient engagement alongside ongoing guidance and monitoring from their provider.

The present patient was a recreational athlete with prior home-based training experience; however, others may need guidance on warm-up, cool-down, and symptom recognition such as dizziness, chest pain, or shortness of breath. Plans should align with medical history, lifestyle factors, and personal goals, progressing gradually with regular imaging to monitor aortic stability. Adherence and safety require active patient-provider collaboration through SDM, emphasizing gradual progression and adherence to exercise parameters such as BP, HR, and RPE thresholds. Routine imaging ensures aortic stability and detects any progression or aneurysmal dilatation, informing necessary adjustments to ExT plans.

## Conclusions

Individualized low-intensity exercise programs with gradual progression show potential for safe rehabilitation in patients after AD. The present case demonstrates that moderate exercise, with continuous follow-up, can help manage comorbidities such as hypertension and obesity. After 15 months of base training, aerobic intervals were safely integrated. Resting systolic BP decreased by 13%, with subjective improvements in exercise tolerance and mental well-being, without complications. However, long-term durability remains uncertain. Further research should evaluate the safety and efficacy of structured exercise in this population, emphasizing routine imaging to ensure aortic stability and detect any progression, recurrent events, or complications associated with exercise.

## Funding Support and Author Disclosures

The authors have reported that they have no relationships relevant to the contents of this paper to disclose.
